# Comparison of Chain-Length Preferences and Glucan Specificities of Isoamylase-Type α-Glucan Debranching Enzymes from Rice, Cyanobacteria, and Bacteria

**DOI:** 10.1371/journal.pone.0157020

**Published:** 2016-06-16

**Authors:** Taiki Kobayashi, Satoshi Sasaki, Yoshinori Utsumi, Naoko Fujita, Kazuhiro Umeda, Takayuki Sawada, Akiko Kubo, Jun-ichi Abe, Christophe Colleoni, Steven Ball, Yasunori Nakamura

**Affiliations:** 1 Faculty of Bioresource Sciences, Akita Prefectural University, Shimoshinjo-Nakano, Akita, Japan; 2 Faculty of Agriculture, Kagoshima University, Kagoshima, Japan; 3 Unité de Glycobiologie Structurale et Fonctionnelle, Université des Sciences et Technologies de Lille, Villeneuve d’Ascq, France; 4 Akita Natural Science Laboratory, Tennoh, Katagami, Akita, Japan; University of Insubria, ITALY

## Abstract

It has been believed that isoamylase (ISA)-type α-glucan debranching enzymes (DBEs) play crucial roles not only in α-glucan degradation but also in the biosynthesis by affecting the structure of glucans, although molecular basis on distinct roles of the individual DBEs has not fully understood. In an attempt to relate the roles of DBEs to their chain-length specificities, we analyzed the chain-length distribution of DBE enzymatic reaction products by using purified DBEs from various sources including rice, cyanobacteria, and bacteria. When DBEs were incubated with phytoglycogen, their chain-length specificities were divided into three groups. First, rice endosperm ISA3 (OsISA3) and *Eschericia coli* GlgX (EcoGlgX) almost exclusively debranched chains having degree of polymerization (DP) of 3 and 4. Second, OsISA1, *Pseudomonas amyloderamosa* ISA (PsaISA), and rice pullulanase (OsPUL) could debranch a wide range of chains of DP≧3. Third, both cyanobacteria ISAs, *Cyanothece* ATCC 51142 ISA (CytISA) and *Synechococcus elongatus* PCC7942 ISA (ScoISA), showed the intermediate chain-length preference, because they removed chains of mainly DP3-4 and DP3-6, respectively, while they could also react to chains of DP5-10 and 7–13 to some extent, respectively. In contrast, all these ISAs were reactive to various chains when incubated with amylopectin. In addition to a great variation in chain-length preferences among various ISAs, their activities greatly differed depending on a variety of glucans. Most strikingly, cyannobacteria ISAs could attack branch points of pullulan to a lesser extent although no such activity was found in OsISA1, OsISA3, EcoGlgX, and PsaISA. Thus, the present study shows the high possibility that varied chain-length specificities of ISA-type DBEs among sources and isozymes are responsible for their distinct functions in glucan metabolism.

## Introduction

α-Glucan debranching enzymes (DBE) cleave the α-1,6 glucosidic linkages of branched α-glucans such as amylopectin and glycogen. It is generally accepted that plant DBEs play important roles in a complete starch degradation by cleaving the α-1,6 glucosidic linkages because other enzymes involved in starch breakdown such as α-amylase, β-amylase, plastidial α-glucan phosphorylase (Pho1), and plastidial disproportionating enzyme (DPE1) are capable of hydrolysis and/or transfer of the α-1,4 glucosidic linkages, but not the α-1,6 glucosidic linkages. Higher plant DBEs are composed of isoamylase (ISA, EC 3.2.1.68) and pullulanase (PUL, EC 3.2.1.41), and they have usually four DBE isozymes; three ISA isozymes, ISA1, ISA2, and ISA3 and one PUL [1]. ISA and PUL have distinct glucan specificities. PUL can split the maltotriosyl (DP2) chains of glucans and debranch pullulan while ISA is unable to react to the DP2 chains and pullulan [2,3].

The distinct fine structure of amylopectin has been characterized by the fact that it is composed of the tandem-linked clusters, a structural unit of amylopectin, with a fixed size of approximately 9 nm [4–6], while glycogen has no such structural features. The involvements of DBEs in amylopectin synthesis have been proposed from extensive studies with maize [7–12], rice [13–19], potato [20,21], barley [22], *Arabidopsis* [23–29], *Chlamydomonas* [30–33], and a *Cyanobacterium* sp. CLg-1 [34]. The role of DBE, especially ISA-type, is considered to trim the cluster structure by clearing the ill-positioned branches of the precursor glucans generated by BEs so that otherwise such branches would interfere the formation of ordered double helices between neighboring side chains [1,35–38]. Past biochemical and molecular studies also supported that ISA1 plays an essential role in starch biosynthesis and PUL supports the role of ISA1 to some extent at least in some cereal endosperm, whereas ISA3 is involved in catabolism of starch in plant tissues [35].

Thus, DBEs play different roles in starch biosynthesis and/or degradation. At the same time it is known that there is a large variation in the fine structure of storage α-glucans such as amylopectin, semi-amylopectin, and glycogen in plants, red algae, and cyanobacteria [39–42]. Therefore, it is highly possible that DBEs influence the α-glucan fine structure.

To achieve insights into the molecular basis for roles of DBEs in glucan metabolism, in the present study we attempted to analyze comprehensively the chain-length preference of DBEs, mainly ISA-type DBEs, from various organisms including rice, cyanobacteria, and eubacteria. As higher plant DBEs, rice ISA1 (OsISA1), ISA3 (OsISA3), and pullulanase (OsPUL) were used. Among cyanobacterial DBEs, we selected *Cyanothece* ISA (CytISA), a semi-amylopectin producing cyanobacterium [43], and *Synechococcus elongatus* PCC 7942 (ScoISA) and *Synechocystis* sp. PCC6803 DBEs (slr0237 and slr1857), both of which are known to produce glycogen [39]. For bacterial ISA-type DBEs, *Escherichia coli* GlgX (EcoGlgX) and *Pseudomonas amyloderamosa* ISA (PsaISA) were examined because it is known that the activity of the former is very specific for short chains of glucans [44] while the latter can attack any chains having different chain-lengths [45]. The present study established a great variation in chain-length specificities among DBEs when amylopectin and phytoglycogen were used in in vitro analyses, and the relationship between their specificities and metabolic roles are also discussed.

## Materials and Methods

### Reagents

Glucose and maltose, maltotriose, and maltotetraose and maltohexaose were purchased from Wako Pure Chemical Industries, Ltd. (Tokyo), SIGMA, and Hayashibara Biochemical Laboratories Inc. (Okayama, Japan). Pullulan and red-pullulan were obtained from Hayashibara Biochemical Laboratories Inc. and Megazyme Inc., respectively. Phosphorylase a from rabbit liver and β-amylase from barley were purchased from SIGMA and Biocon Ltd. (Nagoya, Japan), respectively.

### Preparation of α-glucans

The amylopectin from endosperm from japonica rice cultivar Nipponbare was removed from amylose which was precipitated with 1-butanol and isoamylalcohol according to the method of Takeda et al [46]. To remove the lower molecular weight-glucans in the starch preparation, 20 mg of these preparations were dissolved in 1 mL of 100% dimethylsulfoxide (DMSO) at 80°C and added to 8 mL of distilled water. The dissolved amylose was precipitated by adding 1 mL of 100% 1-butanol and 100 μL of 5 M NaCl, and centrifuged at 10,000×*g* for 20 min. The amylopectin-rich solution was added by 4 volumes of ethanol and stand for overnight at 4°C. The glucans in the solution were precipitated by centrifugation at 10,000×*g* for 20 min, and the precipitate was resolved in 1 mL of DMSO. The procedure to remove amylose from amylopectin was repeated 4 times. The finally resulting amylose-free amylopectin precipitate was dried by washing with acetone and diethyl ether. Phytogylcogen from ISA1-deficient *sugary-1* mutant line EM914 isolated from japonica rice cultivar Kinmaze [14] was prepared as described previously [47,48]. Both glucans were stored at room temperature under desiccated condition until use. The phosphorylase a limit dextrin (Φ-LD) and β-amylase limit dextrin (β-LD) were prepared from rice endosperm amylopectin of the *waxy* mutant line EM21, as described previously [42,49].

### Construction and induction of recombinant DBEs

The DNA constructs for recombinant enzymes of rice DBEs (OsISA1 [50], OsISA3 [16], and OsPUL [51]), *Cyanothece* ATCC 51142 ISA (CytISA) [43], *Synechococcus elongatus* PCC 7942 ISA (ScoISA) [52], *Escherichia coli* GlgX (EcoGlgX) [53] were prepared and expressed in *Escherichia coli*, according to the same procedure as described previously [34,54].

The DNA fragment of the *Synechocystis* sp. PCC6803 ISA-type DBE slr0237 (slr0237) [55] was amplified by the polymerase chain reaction (PCR) from genomic DNA of *Synechocystis* sp. PCC6803 strain using the forward primer (5’-CATATGCCACAGTTGATATCTGTTCC-3’) containing NdeI sites and the reverse primer (5’-GTCGACCTATTTTGCCATTAACACCAC-3’) containing the SalI site. The DNA fragment encoding the ISA-type DBE slr1857 (slr1857) from the *Synechocystis* sp. PCC6803 [55] was isolated by amplification using the polymerase chain reaction (PCR) from genomic DNA of *Synechocystis* sp. PCC6803 strain using the forward primer (5’-CATATGGAACGCATAGATATTC-3’) containing NdeI sites and the reverse primer (5’-GTCGACCTATTTTGCCATTAACACCAC-3’) containing the SalI site. The PCRs for amplification of both genes were carried out according to manual protocol of KOD-Plus- DNA polymerase (Toyobo). After addition of 3' A to PCR products by A-attachment Mix (Toyobo), the DNA fragment was subcloned into pGEM-T easy vector. The DNA fragment encoding the slr0237 and slr1857 in the pGEM-T easy vector was excised by restriction enzymes NdeI and SalI. The each DNA fragment was independently ligated with the pCold TF (Takara) expression vector treated with NdeI and SalI. These recombinant DBEs were expressed in BL21 as described previously [19].

### Purification of α-glucan debranching enzyme

All the procedures were performed at 0°C unless otherwise stated. The frozen cells per liter of the above culture were suspended in 50 mL of Medium A including 50 mM imidazol-HCl (pH7.4), 8 mM MgCl_2_, and 1 mM DTT, and 12.5% glycerol. The cells were lysed by sonication and the lysed cells were centrifuged twice at 10,000×*g* for 30 min. The supernatant was applied to a HitrapQ HP (GE Healthcare, Little Chalfont, UK) anion exchange column (5 mL) which had been equilibrated with Medium A. The protein was eluted with a linear gradient of 0–0.5 M NaCl for 30 min at a flow rate of 2 mL min^-1^. The peak fraction of each DBE activity was desalted and concentrated to 0.6 mL by a Centricon 50 centrifugal concentrator (Millipore, Billerica, MA, USA). The concentrated mixture (4 mL) was applied to HPLC using a TSKgel DEAE-5PW column (7.5 mm in diameter × 75 mm in length, Tosoh Corporation, Tokyo, Japan), which had been equilibrated with Medium A. The proteins were eluted with a linear gradient of NaCl concentration (0–0.5 M) in Medium A for 60 min at a flow rate of 1 mL min^-1^ at room temperature. The DBE activity in each fraction was detected with a modification of the bicinchonic acid (BCA) method of Utsumi et al. [54], as described below. The peak fraction of DBE activity was desalted and concentrated as described above and stored at -80°C until use. By these procedures, DBE preparations were purified to a near homogeneity (S1 Fig), which were free from any other hydrolytic activities and were used for further experiments to characterize DBEs.

### DBE enzymatic reactions for characterization of their chain-length preferences

The reaction mixture routinely contained 50 mM Hepes-NaOH buffer (pH 7.4), phytoglycogen (2 mg, equivalent to 982 nmole chains) or amylopectin (2 mg, equivalent to 566 nmole chains), and DBE in a total volume of 400 μL. The reaction was run at 30°C. The amounts of DBEs used were 0.13 μg or 0.13 μg for OsISA1, 32 μg or 9.54 μg for OsISA3, 32 μg or 32 μg for CytISA, 30.3 μg or 30.3 μg for ScoISA, 32 μg or 32 μg for EcoGlgX, and 0.11 μg or 0.11 μg for PsaISA, respectively when the enzyme was incubated with phytoglycogen or amylopectin, respectively. At appropriate intervals, the enzymatic reaction was terminated by heating the reaction mixture in a boiling water for 5 min.

### DBE enzymatic reactions for determination of activities towards various glucans

The reaction mixture routinely contained 50 mM Hepes-NaOH buffer (pH 7.4), DBE, and glucan substrate (0.5 mg), namely amylopectin, β-LD, Ф-LD, glycogen from oyster, or pullulan in a total volume of 100 μL. The reaction was run at 30°C. Assays were conducted in the range of concentrations of purified enzyme where the activity determined as the average of three measurements increased with increases in the reaction time. The enzymatic activities were measured by the BCA method of Utsumi et al. [54], as described below.

### Other DBE enzymatic reactions

In an attempt to clarify the specificity of DBEs for very short side chains of Ф-LD (DP4) and β-LD (DP2 and 3) prepared from rice amylopectin, enzymatic reactions were conducted in 50 mM Hepes-NaOH buffer (pH 7.4), 0.5 mg Ф-LD or β-LD, and the purified DBE preparation in a total volume of 100 μL. The amounts of DBE used were 0.13 μg for OsISA1, 3–5 μg for OsISA3, 1–3 μg for CytISA, 3–6 μg for ScoISA, and 2–4 μg for EcoGlgX, respectively. The reaction was run at 30°C for 60 min and terminated by heating the mixture at 100°C for 5 min. To detect the liberated chains from pullulan by DBEs, the enzymatic reactions were conducted in the same conditions as above.

### Assay of DBE by the BCA method

Assay of DBE was conducted according to the BCA method of Utsumi et al. [54]. Solution A consisted of 97.1 mg of disodium 2,2’-bicinchoninate (BCA), 3.2 g of sodium carbonate monohydrate and 1.2 g of sodium bicarbonate in a total volume of 50 mL. Solution B consisted of 62 mg of copper sulfate pentahydrate and 63 mg of L-serine in a total volume of 50 mL. The working reagent was freshly prepared by mixing equal volumes of the Solutions A and B [56]. Two hundred and twenty five μL of the carbohydrate sample were added to 225 μL of the working reagent in an Eppendorf tube, and the mixture was incubated at 80°C for 40 min in a water bath. After the incubation, the assay mixture was then cooled to room temperature and stood for 10 min. An aliquot (150 μL) of the BCA treated sample was taken and its absorbance at 560 nm was measured by using the microplate spectrophotometer (Bio-Rad). The absorbance at 560 nm was found to be proportional to the concentration of maltose or glucose in the range of 0 to at least 25 μM of the assay mixture. Each mean value was obtained from the average value of at least three replicate measurements.

### Analysis of chain-length distribution of glucans

The glucans in the rest of the reaction mixture were debranched by incubating in the mixture containing 40 mM Na-acetate buffer (pH 4.4), *Pseudomonas amyloderamosa* isoamylase (PaISA, 2 units, Hayashibara Biochem. Lab., Okayama, Japan), and *Klebsiella pneumonia* pullulanase (0.9 units; Wako, Tokyo, Japan) at 37°C overnight in a total volume of 0.3 mL. The glucan chain-length distribution analysis of the resulting mixture was conducted after labeling of the glucans with the fluorescent probe 8-amino-1,3,6-pyrenetrisulfonic acid at their reducing ends according to the fluorophore-assisted carbohydrate electrophoresis (FACE) method reported by O’Shea et al. [57], by using capillary electrophoresis equipped with a laser-induced fluorescence detector (P/ACE MDQ Carbohydrate System, Beckman Coulter/AB SCINEX).

### Analysis of liberated short chains after the DBE enzymatic reactions with Ф-LD, β-LD, and pullulan

Aliquots (20 μL) of the heated enzymatic reaction mixture were applied onto HPLC gel filtration columns composed of a TSKgel G3000PW_XL_ column (7.5 mm in diameter and 30 cm in length) and two TSKgel G-Oligo-PW columns (7.5 mm in diameter and 30 cm in length), which had been kept at 60°C. The chromatography was conducted at a flow rate of 0.4 mL distilled water/min, and the contents of carbohydrates and sugars were measured by an Evaporating Light Scattering Detector (Model 300s; SofTA Corporation, CO., U.S.A.). Each sugar and malto-oligosaccharide (MOS) was identified by comparing its elution time with those of individual standard sugars and MOS.

### Measurement of protein amount

Protein concentration was determined by measuring the absorbance at 280 nm in a NanoDrop ND-1000 (NanoDrop Technologies, Inc.).

### Preparation of phylogenetic tree of DBEs

The molecular phylogenetic analysis was performed by using the Maximum Likelihood method based on the JTT matrix-based model in MEGA6 [58]. There were a total of 214 positions in the final dataset and 1000 bootstrap replicates were included. The percentage of trees in which the associated taxa clustered together is shown next to the branches.

## Results

### Chain preference of DBEs toward phytoglycogen and amylopectin

The amounts of liberated chains were quantitatively measured by the FACE method [57] after the enzymatic reaction between DBE and phytoglycogen or amylopectin. S2 Fig shows the differences of relative amounts of individual side chains between amylopectin and phytogylcogen used as substrate. The shortest chain of amylopectin had degree of polymerization (DP) 6, whereas that of phytoglycogen was DP3 and it had also short chains of DP4 and DP5. The most abundant chains were DP11 for amylopectin and DP10 for phytoglycogen, respectively. The amounts of long chains of DP≧37 were very low in phytoglycogen due to the lack of clusters which is the structural unit of amylopectin [4,5].

The enzymatic reactions of DBEs from various sources using phytoglycogen or amylopectin as substrate were conducted in the reaction mixture of 100 μL at 30°C, as described in the Methods. Aliquots of the reaction mixture were taken at appropriate time intervals from 5 to 60 min. The amounts of liberated chains from the substrate were measured by the BCA method and the chain-length distribution of the liberated chains was analyzed by the FACE method to examine the chain-length preferences of DBEs.

**Fig 1:**
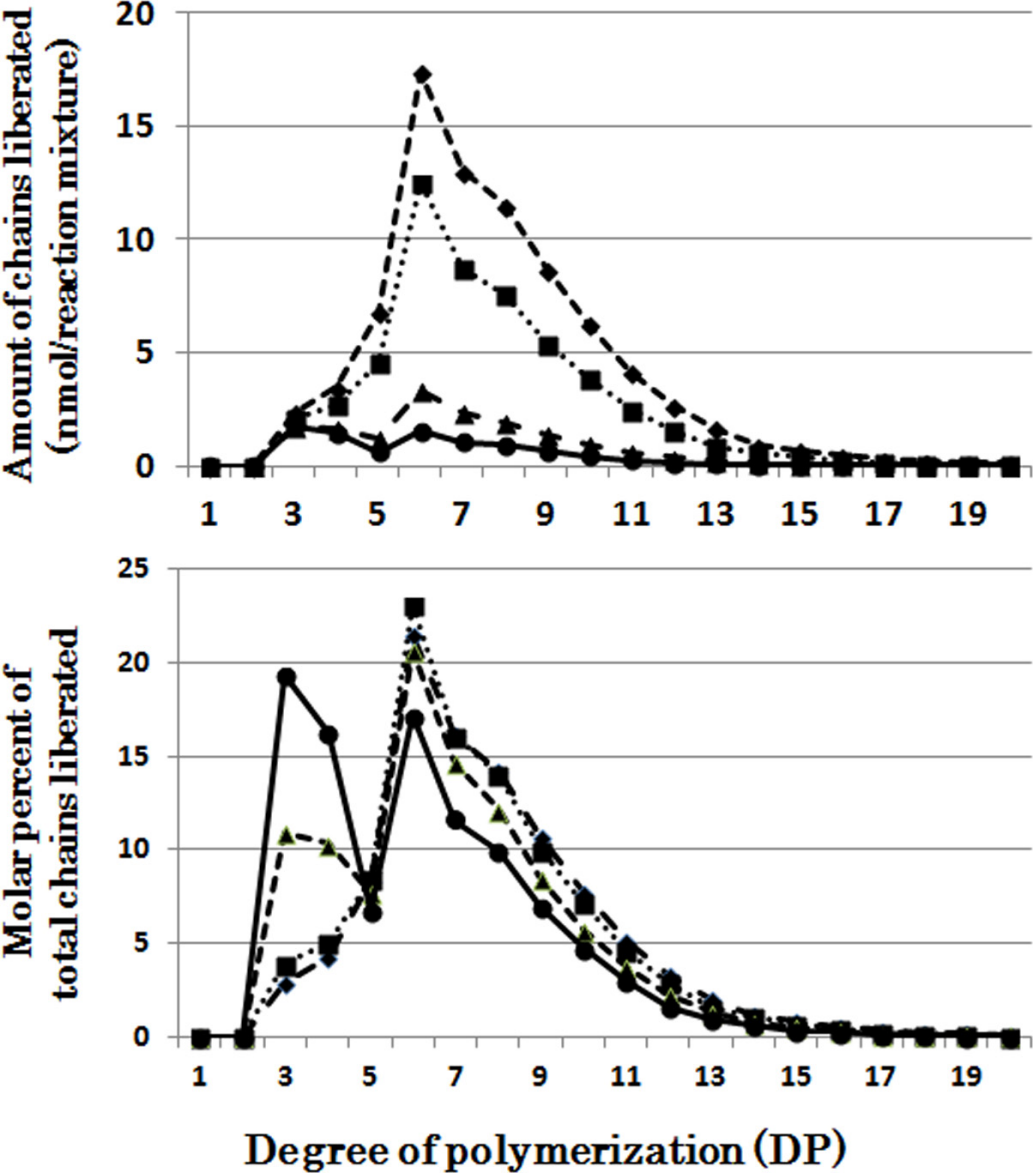
Chain-length preference of rice isoamylase1 (OsISA1) in debranching phytoglycogen. Upper panel shows the amount of chains liberated after enzymatic reaction with OsISA1 and phytoglycogen for 5 (●), 10 (▲), 30 (■), and 60 (♦) min, respectively. Lower panel shows the molar percentage of each liberated chain to the total liberated chains.

**Fig 2:**
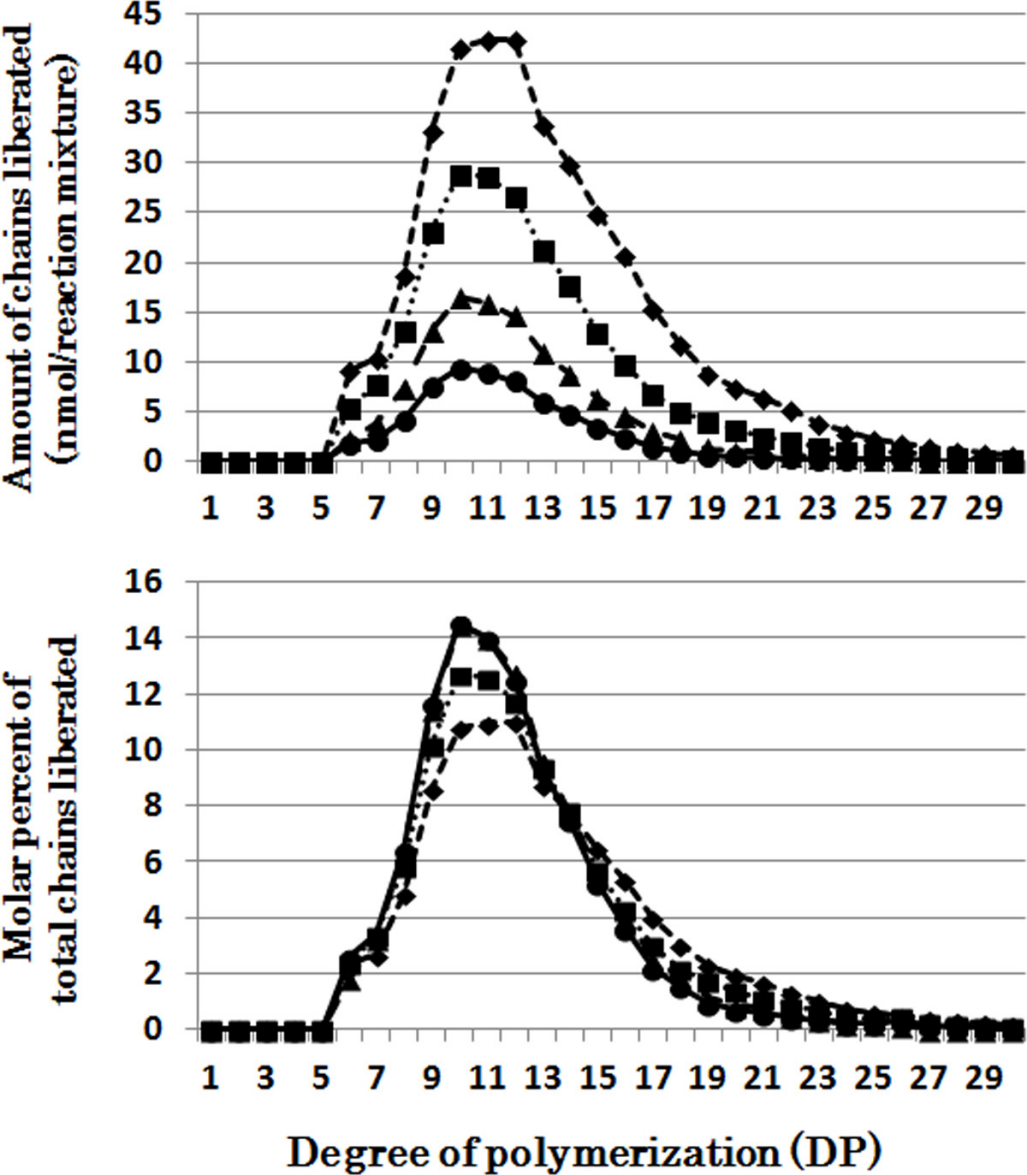
Chain-length preference of rice isoamylase1 (OsISA1) in debranching amylopectin. Upper panel shows the amount of chains liberated after enzymatic reaction with OsISA1 and amylopectin for 5 (●), 10 (▲), 30 (■), and 60 (♦) min, respectively. Lower panel shows the molar percentage of each liberated chain to the total liberated chains.

When OsISA1 was incubated with phytoglycogen, short chains of DP3 and DP4 were most effectively liberated during the short period, and with the elapse of the incubation time intermediate chains with the peak of DP6 were more significantly produced (Fig 1). OsISA1 removed the wide range of chains with DP6-20 from amylopectin although the long chains of DP≧20 were debranched by the prolonged incubation (Fig 2).

**Fig 3:**
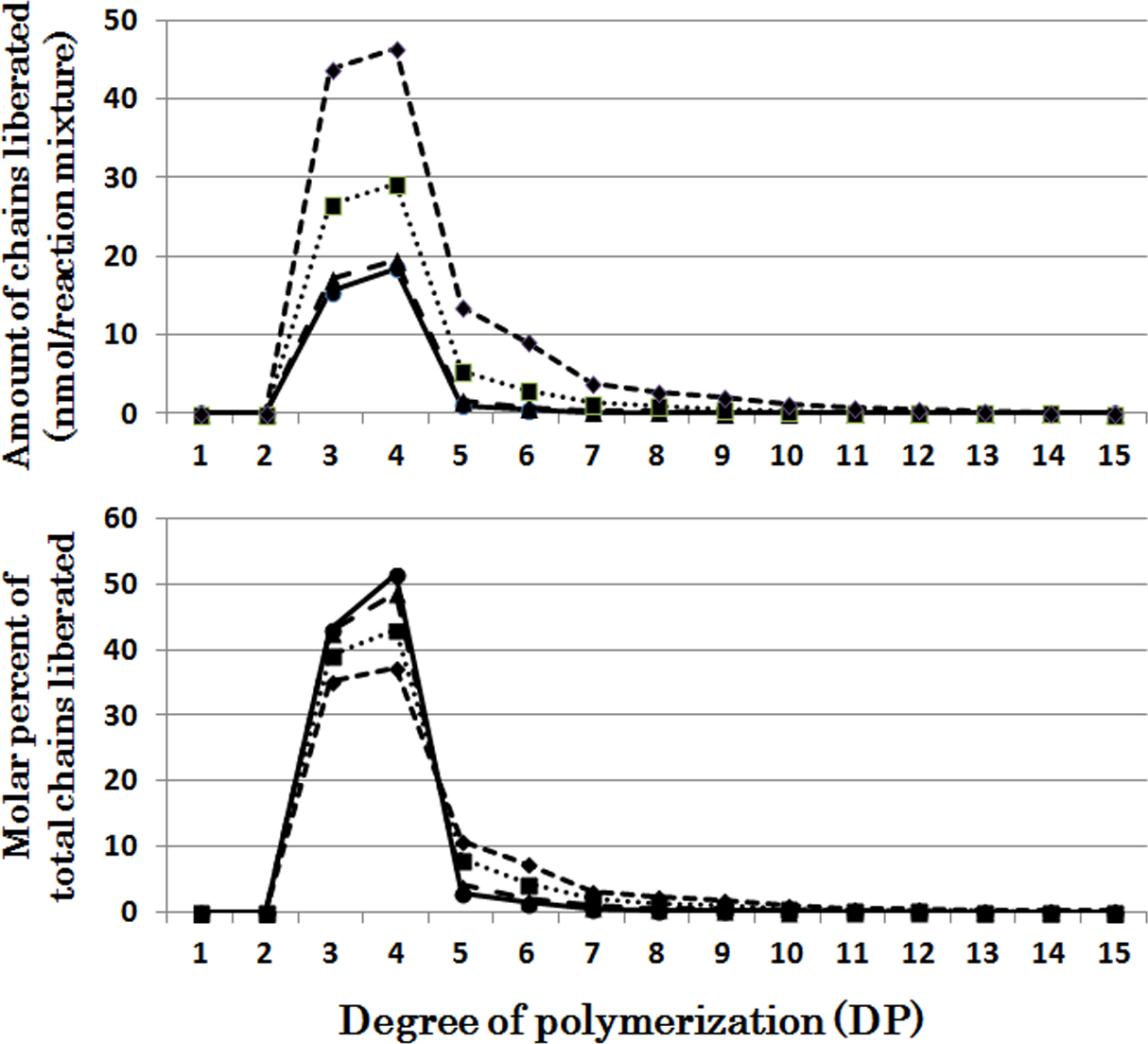
Chain-length preference of rice isoamylase3 (OsISA3) in debranching phytoglycogen. Upper panel shows the amount of chains liberated after enzymatic reaction with OsISA3 and phytoglycogen for 5 (●), 10 (▲), 30 (■), and 60 (♦) min, respectively. Lower panel shows the molar percentage of each liberated chain to the total liberated chains.

Fig 3 presents that chains liberated from phytogycogen by OsISA3 were markedly selective because almost only short chains of DP3 and DP4 were found after the OsISA3 reaction for 5–10 min. Then during the course of enzymatic reaction until 60 min a small amount of the DP5-10 chains was found. The chain-length patterns of chains produced after the reaction of OsISA3 with amylopectin were dramatically different from those with phytoglycogen (S3 Fig). OsISA3 could react to every chain with DP ranging from 6 to about 21, and the pattern of liberated chains was unchanged by the incubation time for 5–60 min.

**Fig 4:**
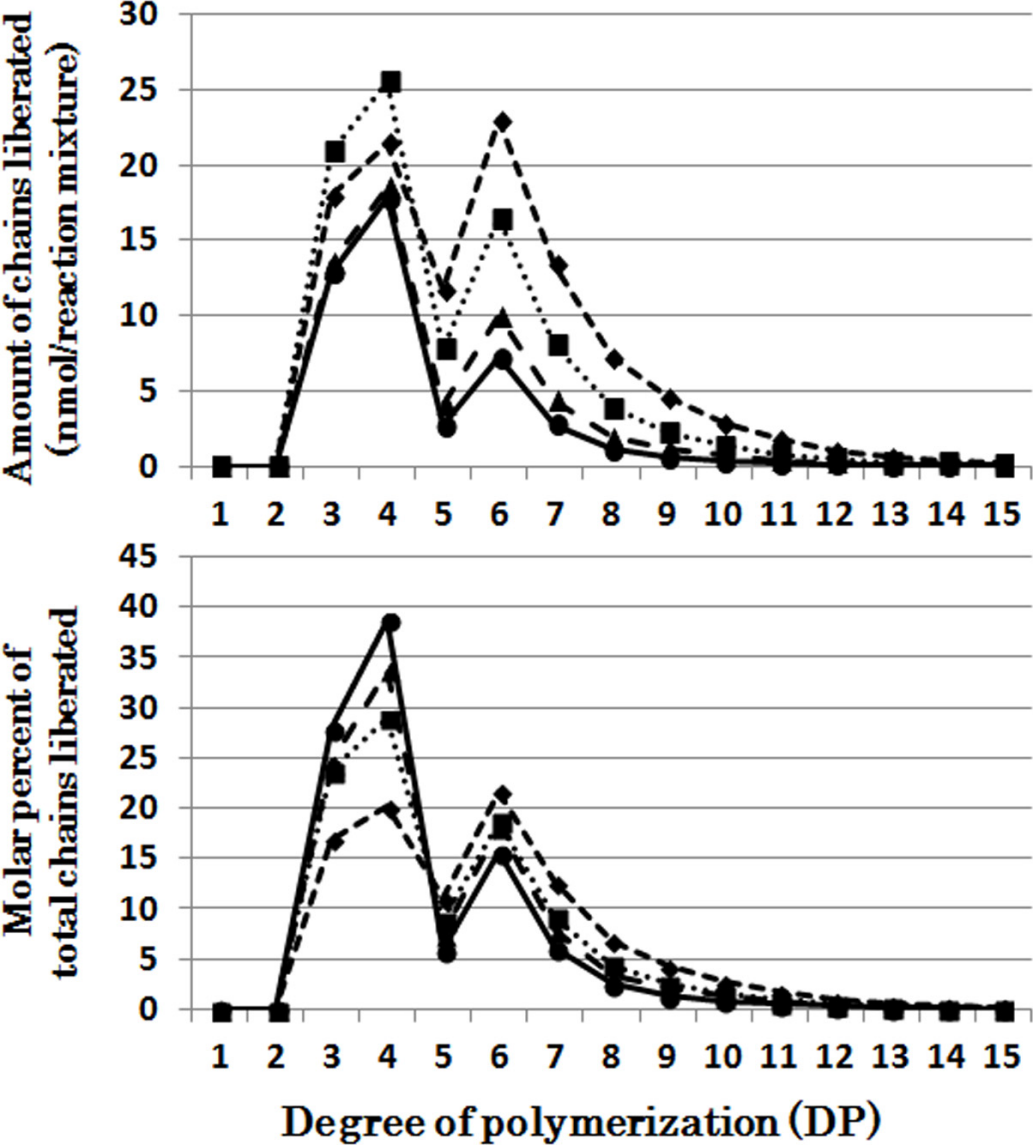
Chain-length preference of Cyanothece ATCC 51142 isoamylase (CytISA) in debranching phytoglycogen. Upper panel shows the amount of chains liberated after enzymatic reaction with CytISA and phytoglycogen for 5 (●), 10 (▲), 30 (■), and 60 (♦) min, respectively. Lower panel shows the molar percentage of each liberated chain to the total liberated chains.

When CytISA was incubated with phytoglycogen, the liberated chains formed two distinct peaks; the first large peak of DP3-4 and the second small peak of DP5-10 (Fig 4), indicating that CytISA could react much more easily to the former chains than the latter chains since that relative amounts of former chains are much lower than the latter chains in phytoglycogen molecules (S2 Fig). It is also noted that although such dual peals were also detected in the OsISA1 products with phytoglycogen, the ratio of molar amounts of the second peak to the first peak was markedly higher in the OsISA1 product (Fig 1) than that in the CytISA products (Fig 4). The results show that OsISA1 can debranch intermediate chains in the second peak more efficiently than CytISA. The patterns of chains formed from amylopectin after the debranching reaction of CytISA were similar to those of OsISA1 with an exception that CytISA could debranch short chains of DP6 and DP7 better than OsISA1 (S4 Fig and Fig 2).

**Fig 5:**
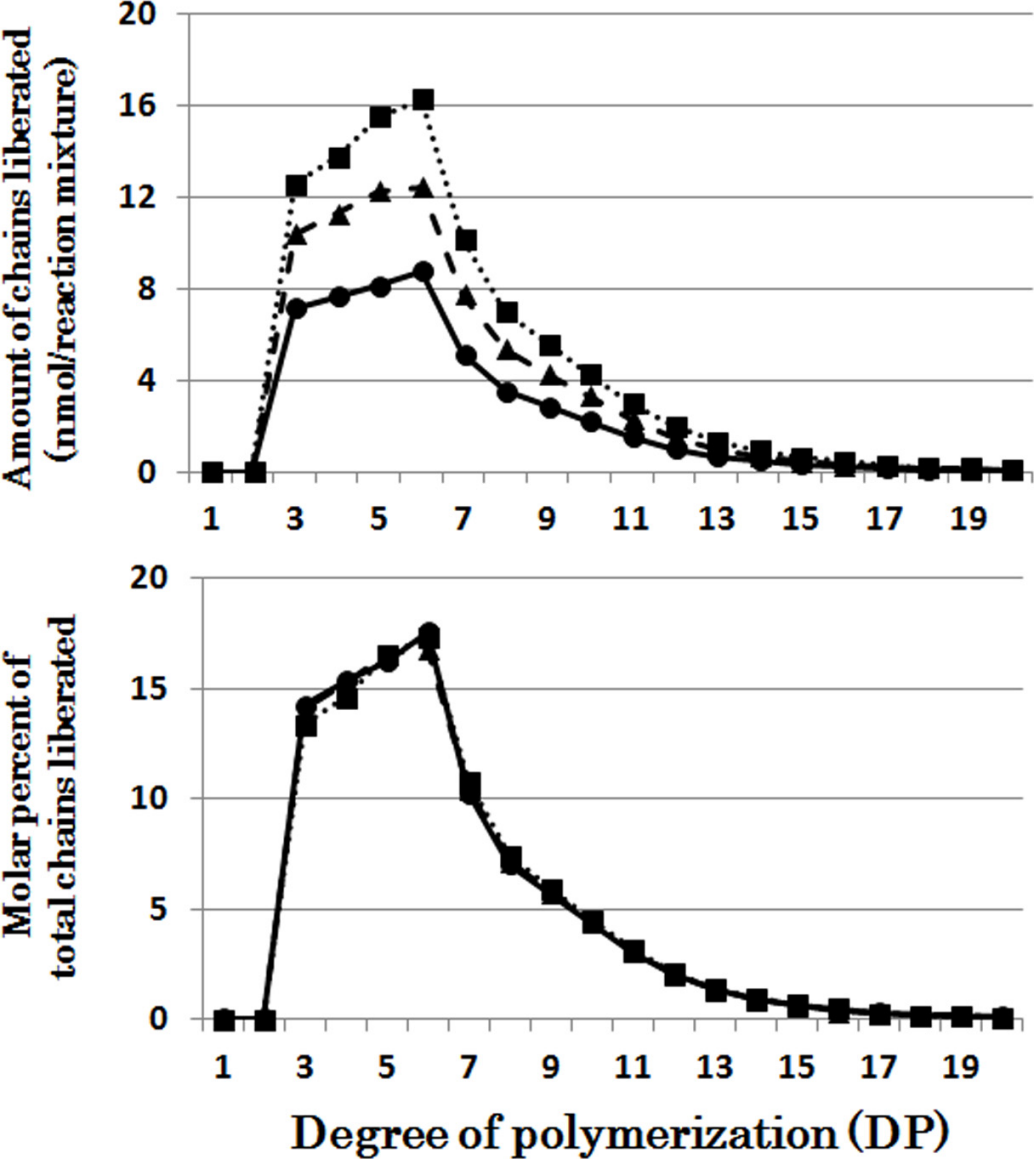
Chain-length preference of *Synechococcus elongatus* PCC7942 isoamylase1 (ScoISA) in debranching phytoglycogen. Upper panel shows the amount of chains liberated after enzymatic reaction with ScoISA and phytoglycogen for 5 (●), 10 (▲), and 30 (■) min, respectively. Lower panel shows the molar percentage of each liberated chain to the total liberated chains.

The liberated chains from phytoglycogen after the ScoISA were enriched in short chains of DP3-6, but contained longer chains of DP≧7, decreasing in amounts with the increase in chain-length until DP about 15 (Fig 5). The chain-length distribution patterns in the ScoISA reaction products with amylopectin used as substrate were similar to those in the OsISA3 products (S5 and S3 Figs).

**Fig 6:**
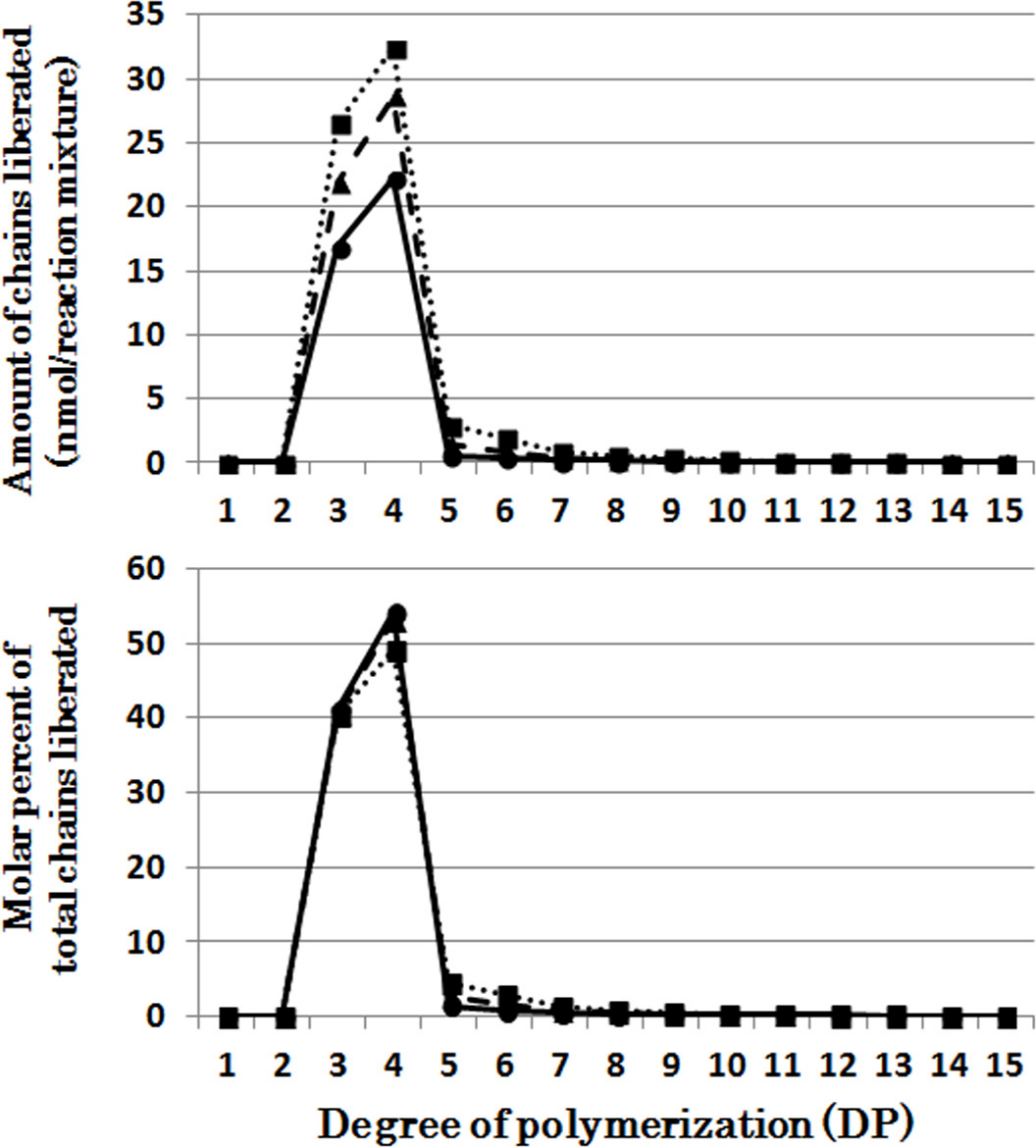
Chain-length preference of *Escherichia coli* GlgX (EcoGlgX) in debranching phytoglycogen. Upper panel shows the amount of chains liberated after enzymatic reaction with EcoGlgX and phytoglycogen for 5 (●), 10 (▲), and 30 (■) min, respectively. Lower panel shows the molar percentage of each liberated chain to the total liberated chains.

It was shown that EcoGlgX removed only exclusively chains of DP3 and DP4 when incubated with phytoglycogen while DP5-7 chains were slightly liberated after the prolonged incubation (Fig 6), consistent with the previous report by Dauvillée et al. [44]. The patterns of products after the EcoGlgX reaction with amylopectin were almost the same as those of the OsISA3 products (S6 and S3 Figs).

**Fig 7:**
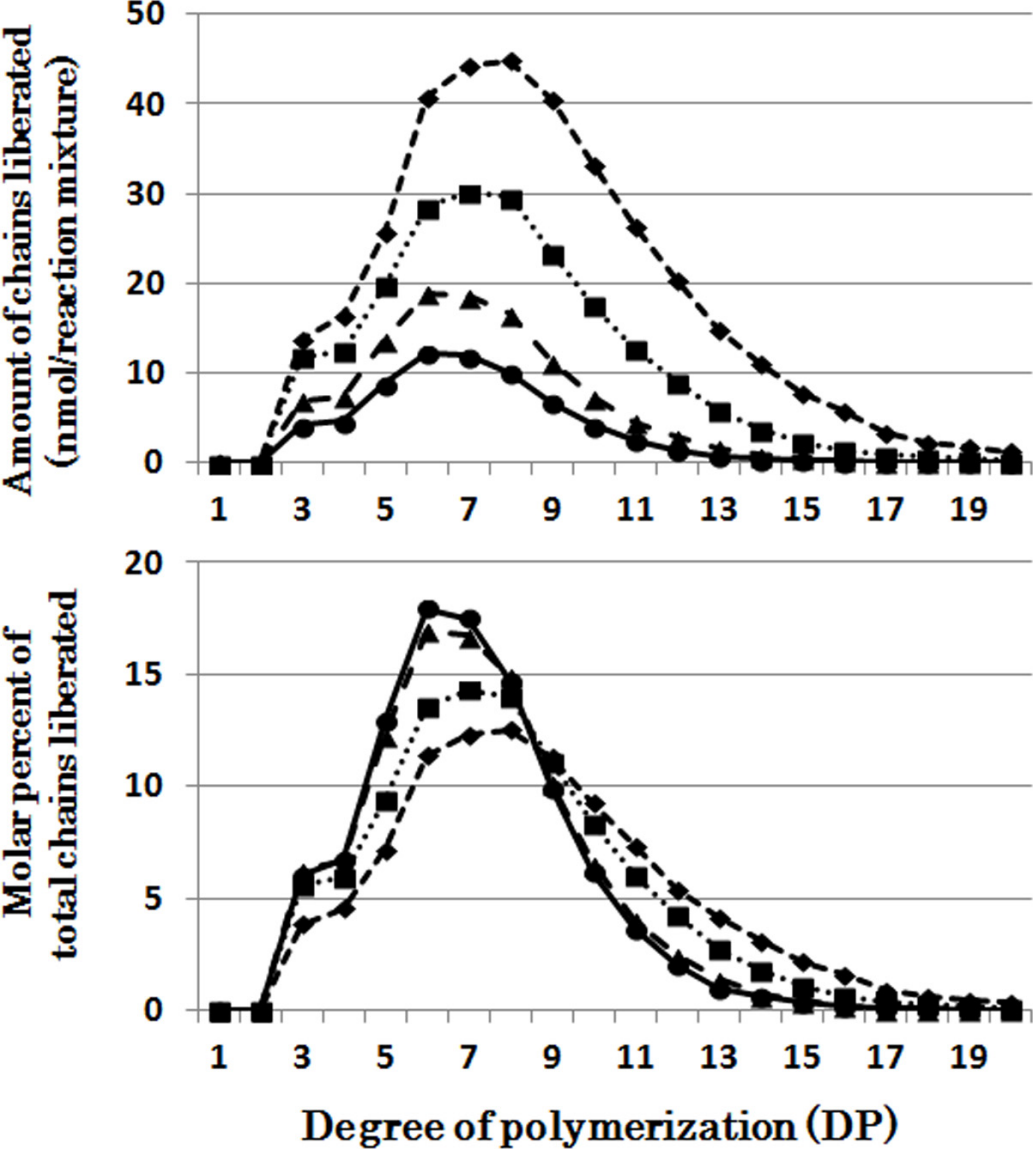
Chain-length preference of *Pseudomonas amyloderamosa* isoamylase (PsaISA) in debranching phytoglycogen. Upper panel shows the amount of chains liberated after enzymatic reaction with PsaISA and phytoglycogen for 5 (●), 10 (▲), 30 (■), and 60 (♦) min, respectively. Lower panel shows the molar percentage of each liberated chain to the total liberated chains.

It is striking that PsaISA could debranch almost all of component chains of phytoglycogen (Fig 7) and amylopectin (S7 Fig).

All these results indicate the great variation of chain-length specificities among ISA-type DBEs from various sources.

### Comparison of chain-length preferences among DBEs

**Fig 8:**
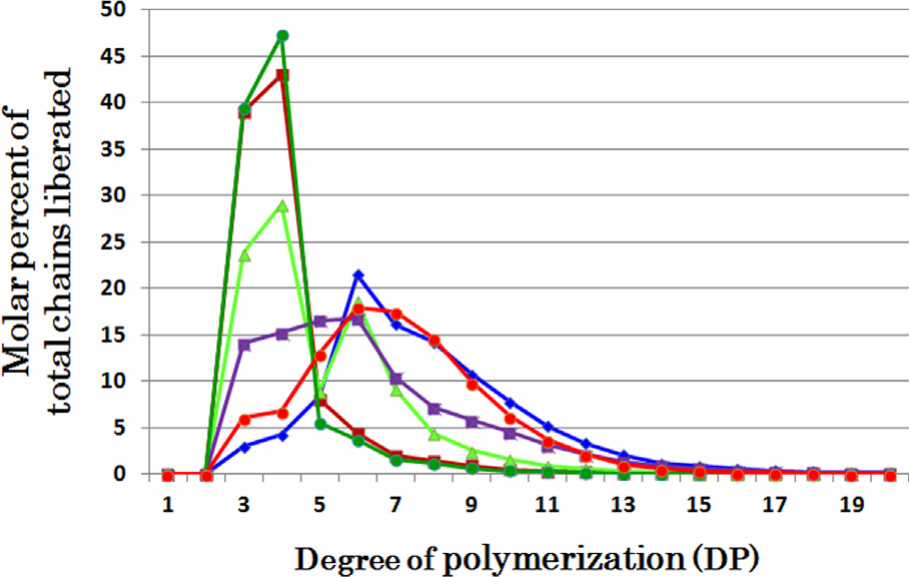
Chain-length preference of ISA-type DBEs in debranching phytoglycogen. The amount of chains liberated after enzymatic reaction with phytoglycogen and OsISA1 (blue ♦), OsISA3 (brown ■), CytISA (green ▲), ScoISA (purple ■), EcoGlgX (dark green ●), and PsaISA (red ●), respectively. The total amounts of liberated chains out of total chains of the substrate glucan phytoglycogen (982 nmoles) by reactions with OsISA1, OsISA3, CytISA, ScoISA, EcoGlgX, and PsaISA were 81, 68, 88, 74, 66, and 68 nmoles, respectively. These data were chosen from Figs 1, 3, 4, 5, 6 and 7, respectively.

The individual DBE activities with phytoglycogen or amylopectin were measured at various time intervals from 5 to 60 min (Figs 1, 2, 3, 4, 5, 6 and 7 and S2, S3, S4, S5, S6 and S7 Figs). To compare the chain-length preferences of DBEs, nearly the same activity levels for various DBEs were chosen. Fig 8 shows that EcoGlgX and OsISA3 reacted remarkably to the very short chains of DP3 and 4 when they were incubated with phytoglycogen. CytISA was also highly reactive to both chains but in addition it also significantly attacked short chains of DP5-10. ScoISA showed the highest activities toward short chains of DP3-6, but it also could react to the longer chains. By contrast, both OsISA1 and PsaISA displayed a broad specificity towards the glucan chain-length of DP≧3 with the maximum activity to chains of DP about 6.

**Fig 9:**
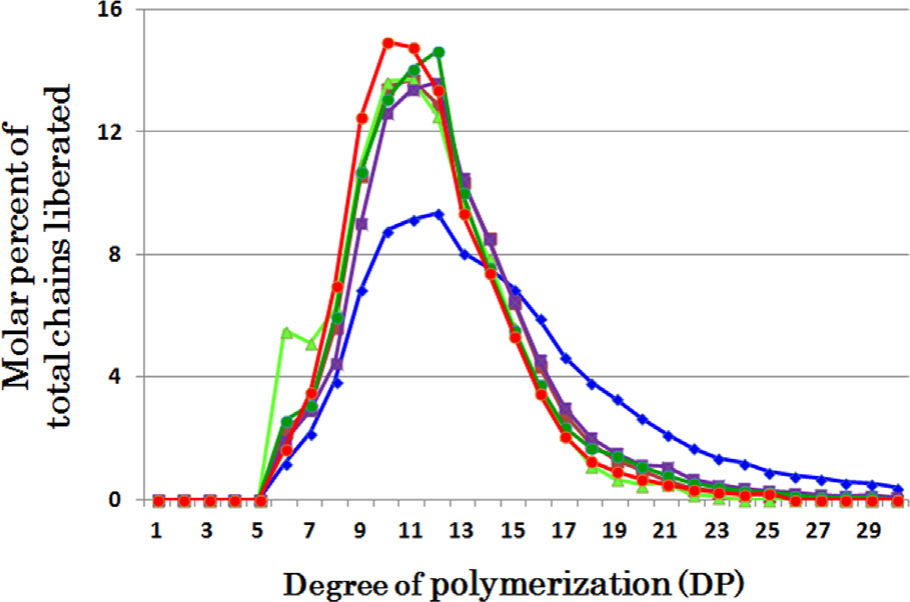
Chain-length preference of isoamylase-type debranching enzymes in debranching of amylopectin. The amount of chains liberated after enzymatic reaction with amylopectin and OsISA1 (blue ♦), OsISA3 (brown ■), CytISA (green ▲), ScoISA (purple ■), EcoGlgX (dark green ●), and PsaISA (red ●), respectively. The total amounts of liberated chains out of total chains of the substrate glucan amylopectin (566 nmoles) by reactions with OsISA1, OsISA3, CytISA, ScoISA, EcoGlgX, and PsaISA were 63, 74, 116, 28, 59, and 89 nmoles, respectively. These data were chosen from Fig 2, S3, S4, S5, S6 and S7 Figs, respectively.

On the other hand, all the DBEs examined could similarly debranch amylopectin chains with a wide range of lengths from DP6 to at least DP20 (Fig 9). Interestingly, however, CytISA reacted markedly to DP6 chains, the minimum chain-length of normal amylopectin external chains (see S2 Fig) and for BE reactions [42,49], while no such preference was found in ScoISA.

### Reactions of DBEs toward pullulan, β-LD, and Ф-LD

It has been reported that ISA and PUL have different glucan specificities [2,3,35]. For example, ISA can highly attack glycogen although PUL does not significantly react with glycogen, while PUL can debranch pullulan, but ISA is inert to pullulan.

**Fig 10:**
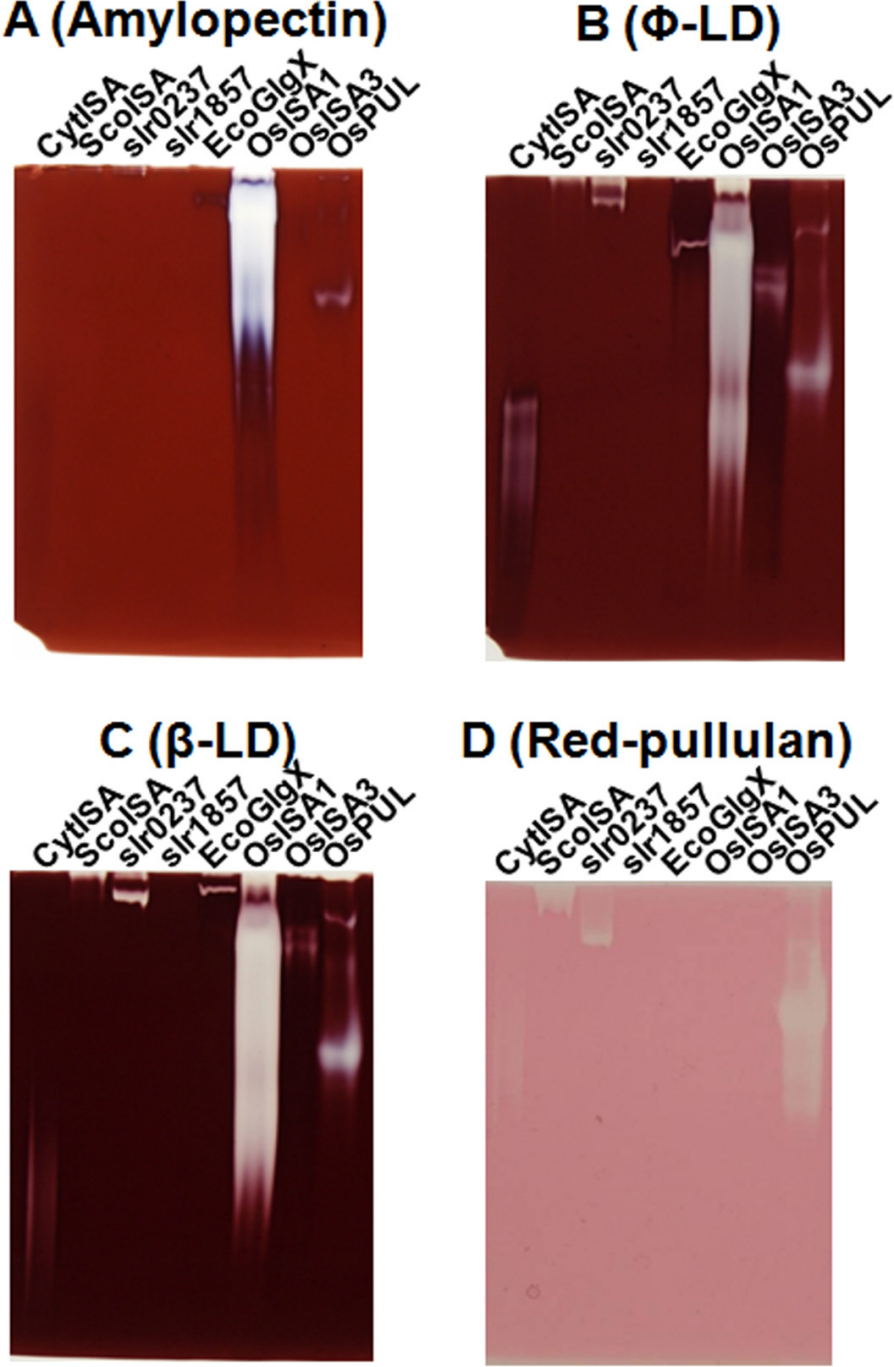
Native-PAGE/staining of activities of DBEs toward amylopetin, Φ-limit dextrins, β-limit dextrins, and red-pullulan. Hydrolytic activities of OsISA1, OsISA3, rice pullulanase (OsPUL), CytISA, ScoISA, *Synechocystis* sp. PCC6803 ISA slr0237, slr1857, and EcoGlgX were visualized by the native PAGE/activity staining method using a polyacrylamide gel including amylopectin (A), phosphorylase a-limit dextrin from rice amylopectin (Φ-LD) (B), β-amylase-limit dextrin from rice amylopectin (β-LD) (C) or red-pullulan (D).

**Fig 11:**
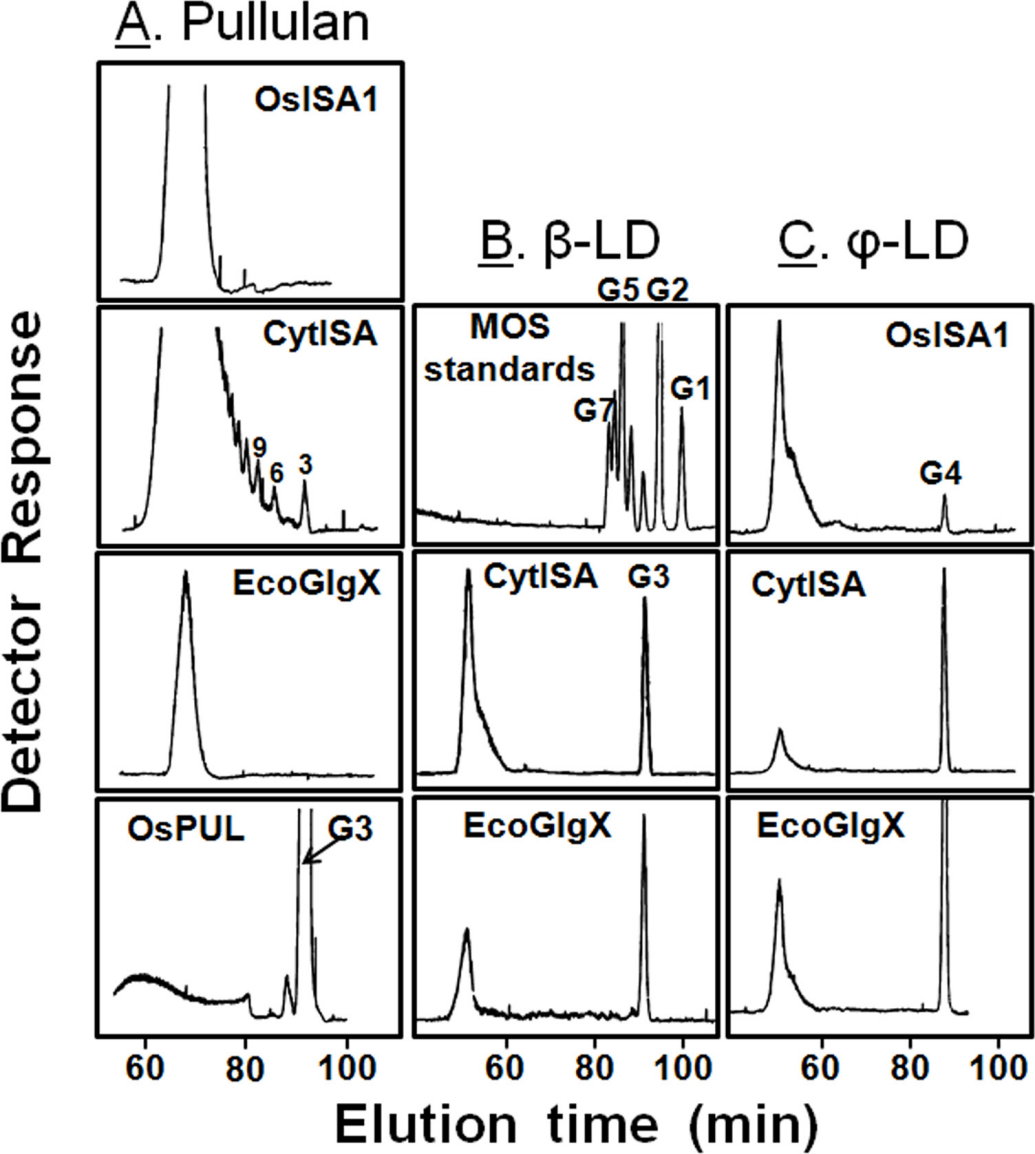
Amounts of MOSs liberated after treatment of pullulan, β-limit dextrins, and Φ-limit dextrins with DBEs. The liberated MOSs liberated after DBE enzymatic reactions with pullulan (A), β-LD (B) or Φ-LD (C) were separated by HPLC gel filtration chromatography and detected by an Evaporating Light Scattering Detector. Sugars or MOS standards: glucose (G1); maltose (G2); maltotriose (G3); maltotetraose (G4); maltopentaose (G5); maltohexaose (G6), and so on.

In this study, the reactions of CytISA, EcoGlgX, and OsISA1 toward pullulan and the chain-preference toward β-LD, and Ф-LD were examined. Firstly, activities of DBEs toward pullulan and other glucans were examined by the zymogram method using native polyacrylamide gel including red-pullulan, amylopectin, Ф-LD of amylopectin or β-LD of amylopectin (Fig 10). CytISA and OsPUL showed the high activity toward pullulan whereas both OsISA1 and OsISA3 were scarcely reactive to pullulan (Fig 10D). Fig 10D also shows that all ISAs from *Cyanothece* ATCC 51142 (CytISA), *Synechococcus elongates* PCC7942 (ScoISA), and *Synechocystis* sp. PCC6803 (slr0237) could significantly attack pullulan. To test further activities of DBEs activities toward pullulan, the reaction mixtures were applied to gel filtration HPLC to detect the presence of maltotriose and maltohexaose, the possible debranched products of pullulan. Fig 11A clearly shows the presence of maltotriose, maltohexaose, maltonanose, and so on, in the CytISA mixture, whereas no such MOSs were formed after the reactions of EcoGlgX, and OsISA1.

Secondly, the capacity of DBE for debranching the DP2 and DP3 chains was examined by using the β-LD of rice amylopectin because the glucan includes maltosyl- and maltotriosyl-chains as its external chain segments [6,59]. Both CytISA and EcoGlgX could liberate only the DP3 chains, but not the DP2 chains, of the β-LD (Fig 11B).

Thirdly, Ф-LD of rice amylopectin with maltotetrasyl-chains as outer segments [6,59] was used for the DBE reaction. Fig 11C shows the formation of the DP4 chains when the Ф-LD was incubated with CytISA, EcoGlgX or OsISA1.

### The activities of DBEs toward various glucans

The substrate glucan specificities of various DBEs were determined by measuring the increase in reducing ends according to the BCA method [56]. Table 1 presents the relative activities of each enzyme toward various glucans including amylopectin, β-LD of amylopectin, Ф-LD of amylopectin, oyster glycogen, and pullulan using the standard activity value as when Ф-LD was used as substrate. Compared with Ф-LD of amylopectin, amylopectin is a better substrate for OsISAS1, whereas no significant activity of OsISA3 was found toward amylopectin. Although both CytISA and EcoGlgX could use all the glucans examined besides pullulan, Ф-LD and β-LD of amylopectin were the best and the second best substrates, respectively. It is noted that among ISA-type DBEs, CytISA was the only one which could significantly react to pullulan. OsPUL was also reactive to a variety of glucans, while pullulan was the best for its activity.

**Table 1:** Relative activities of glucan debranching enzymes toward various branched α-glucans.

α-Glucans	Φ-LD	β-LD	amylopectin	oyster glycogen	pullulan
OsISA1	100	71 ± 5	356 ± 46	104 ± 8	trace
OsISA3	100	48 ± 3	trace	1.5 ± 0.7	trace
CytISA	100	55 ± 4	9.4 ± 2.3	1.4 ± 0.8	2.3 ± 0.7
EcoGlgX	100	34 ± 3	0.8 ± 0.6	2.0 ± 1.5	trace

Each value was the average of at least three replicate measurements. The activities of OsISA1, OsISA3, CytISA, and EcoGlgX toward Φ-LD were 13.5 ± 2.3, 122 ± 17, 5.3 ± 1.1, and 43.3 ± 3.8 nmoles/μg protein/min, respectively.

The results are basically consistent with our previous report [37] that OsISA1, OsISA3, CytISA, ScoISA, and EcoGlgX could debranch side chains of amylopectin and phytoglycogen although their activities toward both glucans greatly differed among DBEs.

## Discussion

### Comparison of preferences of chain-lengths among DBEs

The present studies analyzed in detail the differences in chain-length preferences among mainly 6 DBEs from higher plant (rice, OsISA1 and OsISA3), cyanobacteria (*Cyanothece* sp. ATCC51142, CytISA; *Synechococcus elongatus* PCC7942, ScoISA), and bacteria (*Escherichia coli*, EcoGlgX; *Pseudomonas amyloderamosa*, PsaISA). Table 2 summarizes the chain-length specificities of various DBEs revealed in this study as well as those reported previously by many groups ([44,47,60], and references therein).

**Table 2:** Chain-length specificities of ISA-type DBEs from various sources.

Sources	Chain preference				Reactivity to		Reference
	(PG or Gly*)	(Type)	(Amylopectin)	(Type)	(β-LD)	(Pullulan***)	
**(ISA-Type)**							
OsISA1	Univ. (DP6)	U	Univ. (DP10-12)	U		X	This study
OsISA1	Univ.	U	Univ.	U			[47]
OsISA1-2	Univ.	U	Univ.	U			[47]
PvISA1-2	Univ.* (DP8,9 & 6)	U	Univ. (DP11-13 & 6)	U	DP2,3		[60]
AtISA1-2	Univ.	U	Univ.	U			[61]
ChlISA1	Univ.	U	Univ.	U			[62]
			Univ.	U		Δ	[30]
OsISA3	DP3,4	S	Univ. (DP10,11)	U	DP3	Χ	This study
PvISA3	DP3,4*	S	DP3,4	S	DP2,3		[60]
CytISA	DP3,4 & 5–10	M	Univ. (DP10,11)	U	DP3	Ο	This study
ScoISA	DP3-6 & 7–13	M	Univ. (DP10-12 & 6)	U		Ο	This study
EcoGlgX	DP3,4	S	Univ. (DP10-12)	U		X	This study
	DP2-4**	S				X	[63]
	DP3,4*	S					[44]
					DP3		[64]
PsaISA	Univ. (DP6,7)	U	Univ. (DP10-12)	U	DP3	X	This study
	Univ.*	U	Univ.	U			[65]
	Univ.	U	Univ.	U			[47]
	Univ.* (DP6,7)	U	Univ. (DP11-13 &6)	U	DP2,3		[60]
**(PUL-Type)**							
OsPUL	Univ.	U	Univ.	U	DP2,3	Ο	This study

PG, phytoglycogen; Gly, glycogen; Univ., the universal chain-type; S, the short chain-type; M, the intermediate chain-type; Pv, *Phaseolus vulgaris*; At, *Arabidopsis thaliana*; Chl, *Chlamydomonas reinhardtii*. For other information on abbreviations see the text.

*Glycogen as substrate.

**β-amylase- and phosphorylase a-limit dextrins of glycogen or small branched MOS as substrate.

***The extent of activity: O, significant; Δ, low; X, not detected.

It is striking to note that chain-length specificities of OsISA1 and OsISA3 toward phytoglycogen were distinct from each other. OsISA1 universally hydrolyzed chains with different chain-lengths (Fig 1), consistent with the results with ISA1 or ISA1-2 from kidney bean (*Phaseolus vulgaris* L.) [60], Arabidopsis [61], and *Chlamydomonas reinhardtii* [62]. In contrast, OsISA3 almost exclusively hydrolyzed the very short chains of DP3 and 4 (Fig 3), as found in kidney bean ISA3 (PvISA3) [60] and EcoGlgX [44] toward glycogen. The marked differences between OsISA1 and OsISA3 were similarly seen between PsaISA and EcoGlgX (Figs 6, 7 and 8 and Table 2). Thus, we can refer to OsISA1 and PsaISA as the “U-type” DBE (the universal chain-type) and OsISA3 and EcoGlgX as the “S-type” DBE (the short chain-type), respectively (Table 2). On the other hand, the chain-length specificities of CytISA and ScoISA toward phytoglycogen were the intermediate between U-type and S-type because they preferentially reacted to very short chains DP3-4 and DP3-6, respectively, but at the same time they could also hydrolyze the longer chains (Figs 4, 5 and 8 and Table 2). Therefore, we called both CytISA and ScoISA as the “M-type” (the intermediate chain-type) DBE (Table 2). At the same time, however, it is noted that the chain-length specificities of both cyanobacteria ISAs toward phytoglycogen markedly differed from each other (compare Fig 4 for CytISA with Fig 5 for ScoISA), indicating a large variation in chain-length specificity among cyanobacteria ISAs. In conclusion, ISA-type DBEs so far examined can be classified into 3 groups in terms of chain-specificities to glycogen or phytoglycogen.

We also confirmed that all the six DBEs commonly displayed a universal broad chain-length preference toward amylopectin (the U-type) (Figs 2 and 9, S3, S4, S5, S6 and S7 Figs, and Table 2) in spite of marked variations toward glycogen or phytoglycogen. The results are consistent with our previous report with rice ISA1-homomer and ISA1-ISA2 heteromer toward amylopectin [47], being in sharply contrast with results by Takashima et al. [60] that PvISA3 shows a strong specificity for very short chains of DP3 and DP4 (the S-type) even when amylopectin is used as substrate. The discrepancy between OsISA3 and PvISA3 might be due to the differences in experimental conditions and/or the intrinsic enzymatic properties between both ISA3, although the exact reasons are unknown.

It has been known that one of the differences between ISA-type and PUL-type DBEs are that the latter can easily remove the maltosyl (DP2) chains of β-LD while the former can scarcely hydrolyze the DP2 chains [2,3]. The present results showed that only DP3 chains, but not DP2 chains, were liberated from β-LD by the reaction with OsISA3 or CytISA (Fig 11B). The results are in sharp contrast with the results with PvISA1-ISA2 heteromer and PvISA3 because both enzymes liberate both DP2 and DP3 chains of β-LD [60] (Table 2), although the exact reasons remain unresolved.

### Activities of ISA-type and PUL-type DBEs toward pullulan

In this study, the reactivity of DBEs toward pullulan was examined by detecting MOS liberated from pullulan after the enzymatic reactions. It was proven that the CytISA reaction product contained maltotriose, maltotexaose, and other MOSs of DP3n (Fig 11A), as found in the OsPUL reaction products, indicating that CytISA can react to pullulan. However, no such MOSs were detected in the OsISA1 and EcoGlgX reaction products (Fig 11A). Although Yokobayashi et al. [65] reported that PsaISA possesses a slight activity toward pullulan, no such activity was detected in our experimental conditions (data not shown). Thus, CytISA is a unique DBE with significant catalytic properties of both ISA-type and PUL-type. The slight activities of cyanobacteria ISAs from *Cyanothece* (CytISA), *Synechochoccus* (ScoISA), and *Synechocystis* sp. PCC6803 (slr0237) toward pullulan were also shown by zymogam analysis (Fig 10D), suggesting that cyanobacteria ISAs have commonly some activities toward pullulan.

### Possible roles of DBEs in glucan metabolism

What is the requisite for DBE involved in the starch biosynthetic enzyme? Firstly, it must efficiently cleave the chains of DP≧6 of the intermediate glucans because the minimum chain-length of transferred chains for plant BEs is DP6 [38,42,49]. It is also known that plant BE isoforms such as BEI, BEIIa, and BEIIb display distinct chain-length specificities for transferred chains [42,49]. To respond to such newly branched chains with different chain-lengths, DBE must have universal properties regarding reactivity to chains having a wide range of chain-length. The properties of OsISA1 met such requirement while OsISA3 did not (Figs 1, 2 and 3, S3 Fig, and Table 2).

Secondly, the activity of DBE toward glucans such as amylopectin and phytoglycogen is required to be high so that it plays a role in trimming the shape of glucans. The rate of amylopectin biosynthesis is very high in plant tissues, especially in reserve tissues, and hence the timing of trimming by removing ill-positioned branches is only when new branches are formed by BEs. OsISA1 showed high activities toward amylopectin and phytoglycogen as well as β-LD and Φ-LD from amylopectin whereas OsISA3 had a low activity to amylopectin and phytoglycogen compared with activity to Φ-LD from amylopectin (Table 1 and Cenci et al. [34]).

These results are consistent with an idea that OsISA1, but not OsISA3, can be involved in starch biosynthesis. The role of ISA1 in trimming the shape of the cluster structure of amylopectin constitutes an essential part in starch biosynthesis [8,35–38]. It is considered that ISA1 hydrolyzes more preferably α-1,6 glucosidic linkages which are sparsely present than those which are densely located in glucans. In the starch biosynthetic process, BEs possibly form most of α-1,6 glucosidic linkages within restricted region of the substrate/acceptor glucans whereas they happen to transfer some chains to the acceptor chains remote from the region. The latter chains must be cleared off immediately because these chains will interfere in the formation of double helices, which might be the basis of the ordered cluster structure of amylopectin.

It has been widely accepted that DBEs play crucial roles in starch degradation [35,44]. It is striking to note that ISA3 from broad bean [60] and rice (Fig 3) and *E*. *coli* GlgX (EcoGlgX) protein [44] (Fig 6) are specified to react to only very short chains of DP3-4 in α-glucans. Thus, it is unlikely that these DBEs are involved in the primary reaction of starch degradation because the minimum chain-length of normal amylopectin chains is DP6 and because even in phytoglycogen the most abundant side (A) chains have length of DP6 to up to about 10 (S2 Fig). Instead, as proposed by Dauvillée et al. [44], phosphorylase and β-amylase initiate starch catabolism by attacking side chains to shorten their lengths to DP4 and DP2-3, respectively. The idea meets the results with high chain-length specificity of ISA3 and EcoGlgX because their specific roles in starch catabolism are to clear the very short chains produced by actions of phosphorylase and β-amylase. The observations that OsISA3 and EcoGlgX were almost inactive to amylopectin and glycogen (Table 1) might mean that they are kept inert to mature α-glucans when these compounds are synthesized and stored in cells. It is also considered that PUL-type DBE plays a role in removing the DP2 chains formed by β-amylolysis from side chains of DP having even-number.

In contrast to enterobacteria, cyanobacteria harbor a variable number of DBEs, from 1 to 5, distributed into three families (GlgX/TreX/ISA-type DBE, amylopullulanase and amylo-1,6 glucosidase) [41]. Such diversity in DBE activities might reflect overlapping functions in both glucan catabolism and anabolism.

Recent studies established that some cyanobacteria synthesize starch-type α-glucans (semi-amylopectin [39,41–43,66] and amylose [43]). Cenci et al. [34] reported that all the phytoglycogen accumulating mutants isolated from *Cyanobacterium* sp. CLg-1 are defective in the ISA-type *DBE* gene, indicating that the DBE plays an essential role in the cluster structure of semi-amylopectin. It is also known that *Cyanothece* can synthesize semi-amylopectin [43], while *Synechococcus elongatus* remains to accumulate glycogen-type α-glucans in the cell [39,66]. It is interesting to note whether cyanobacteria ISAs have different properties from EcoGlgX, considering that ISAs from some cyanobacteria play a part in the synthesis of semi-amylopectin. The present study showed that cyanobacteria ISAs such as CytISA and ScoISA have different enzymatic properties from EcoGlgX in several respects. First, although EcoGlgX only debranched chains of DP3 and 4 from phytoglycogen (Fig 6), both CytISA and ScoISA were highly reactive not only to the DP3-4 chains but also the DP5-8 chains (Figs 4 and 5, respectively). Second, both CytISA and ScoISA could attack pullulan chains to some extent, while EcoGlgX was unable to react to pullulan (Fig 10D and Fig 11A). These results show that cyanobacteria ISAs have wider specificities toward branched α-glucans in terms of chain-lengths and their fine structures, and this might enable these ISAs to be involved in modifying the fine structure of α-glucans. Nevertheless, it is interesting that cyanobacteria ISAs are comparatively close to EcoGlgX in the phylogenetic tree (S8 Fig). This sharply contrasts with the observation that a marked difference in the enzymatic properties between EcoGlgX and PsaISA (Table 2) is related to a significant dissimilarity in the amino acid sequence between them (S8 Fig). Song et al. [64] predicted that EcoGlgX possesses a more compact structure as compared with PsaISA and that the variation of the helix α4 structure greatly influences the differences the shape and biophysical features of the cleft in the active site between both proteins. At present, however, it is difficult to rationally relate the distinct specificities of DBEs to their structural features. It was also reported that EcoGlgX exists in monomer [64] whereas CytISA is present as homo-multimer [34], as ISA1 from higher plants and green algae [30,35]. The molecular mechanism for much diversity in enzymatic properties of DBEs remains to be elucidated in the future investigations.

## Conclusions

The present study revealed a great variation in enzymatic properties of ISA-type DBEs among plants, cyanobacteria, and bacteria. The activities towards various glucans having different branch patterns and external chain-lengths such as phytoglycogen, amylopectin, and Ф-LD and β-LD from amylopectin remarkably varied among DBEs. OsISA3 and EcoGlgX were specified to remove very short chains of phytoglycogen because they substantially reacted to the chains of DP3 and 4. In contrast, OsISA1 and PsaISA could remove all phytoglycogen chains. Thus, they were referred to as the short-chain-type (S-type) and the universal-chain-type (U-type), respectively. Although CytISA and ScoISA most preferentially debranched very short chains of DP3-4 and DP3-6 chains, respectively, they also could react to intermediate-size chains. Thus, these cyanobacteria ISAs belong to the intermediate-type (M-type) in terms of responses to chain-lengths. Although ISA is known to be unable to react to pullulan, which is a good substrate for PUL, cyanobacteria ISAs such as CytISA, ScoISA, and slr0237 could significantly hydrolyze the pullulan branch linkages whereas other ISAs such as OsISA1, OsISA3, EcoGlgX, and PsaISA could not. It was also shown that ISAs so far examined only cleaved chains of DP3, but not those of DP2, from β-LD of amylopectin, while OsPUL removed both the DP2 and DP3 chains. All these results strongly suggest the different roles among DBEs in α-glucan metabolism. Rice plants have both types of ISAs; i.e. OsISA1 and OsISA3, playing distinct parts in starch biosynthesis and degradation, respectively. Although *Cyanothece* ATCC 51142 ISA and *Synechococcus elongatus* PCC7942 synthesize semi-amylopectin [43] and glycogen [39], respectively, both ISAs showed the intermediate types in terms of chain-length preference and glucan specificity (pullulan and glycogen, being a preferred substrate for PUL and ISA, respectively [2]). Although some semi-amylopectin producing cyanobacteria such as *Cyanothece* has only a single ISA isoform, it is possible that the specific properties of cyanobacteria ISA could play an essential role in the synthesis of starch-like α-glucans instead of glycogen.

## Supporting Information

S1 Fig SDS-PAGE of purified DBE preparations used in this study. (TIF)

S2 Fig Chain-length distribution of amylopectin and phytoglycogen. Amylopectin from mature endosperm of rice japonica cultivar Nipponbare and phytoglycogen from mature endosperm of rice *sugary-1* mutant line EM 914 [14].
(TIF)

S3 Fig Chain-length preference of rice isoamylase3 (OsISA3) in debranching amylopectin. Upper panel shows the amount of chains liberated after enzymatic reaction with OsISA3 and amylopectin for 5 (●), 10 (▲), 30 (■), and 60 (♦) min, respectively. Lower panel shows the molar percentage of each liberated chain to the total liberated chains.
(TIF)

S4 Fig Chain-length preference of Cyanothece ATCC 51142 isoamylase (CytISA) in debranching amylopectin. Upper panel shows the amount of chains liberated after enzymatic reaction with CytISA and amylopectin for 5 (●), 10 (▲), 30 (■), and 60 (♦) min, respectively. Lower panel shows the molar percentage of each liberated chain to the total liberated chains.
(TIF)

S5 Fig Chain-length preference of *Synechococcus elongatus* PCC7942 isoamylase1 (ScoISA) in debranching amylopectin. Upper panel shows the amount of chains liberated after enzymatic reaction with ScoISA and amylopectin for 5 (●), 10 (▲), 30 (■), and 60 (♦) min, respectively. Lower panel shows the molar percentage of each liberated chain to the total liberated chains.
(TIF)

S6 Fig Chain-length preference of *Escherichia coli* GlgX (EcoGlgX) in debranching amylopectin. Upper panel shows the amount of chains liberated after enzymatic reaction with EcoGlgX and amylopectin for 5 (●), 10 (▲), 30 (■), and 60 (♦) min, respectively. Lower panel shows the molar percentage of each liberated chain to the total liberated chains.
(TIF)

S7 Fig Chain-length preference of *Pseudomonas amyloderamosa* isoamylase (PsaISA) in debranching amylopectin. Upper panel shows the amount of chains liberated after enzymatic reaction with PsaISA and amylopectin for 5 (●), 10 (▲), 30 (■), and 60 (♦) min, respectively. Lower panel shows the molar percentage of each liberated chain to the total liberated chains.
(TIF)

S8 Fig Phylogenetic tree of α-glucan debranching enzymes. DBEs used in this study were shown in the parenthesis.
(TIF)
