# Supplementary figures and images for: Comparison of Chain-Length Preferences and Glucan Specificities of Isoamylase-Type α-Glucan Debranching Enzymes from Rice, Cyanobacteria, and Bacteria
Source: PLoS One. 2016 Jun 16;11(6):e0157020. doi: 10.1371/journal.pone.0157020 (PMC4911114; doi:10.1371/journal.pone.0157020)

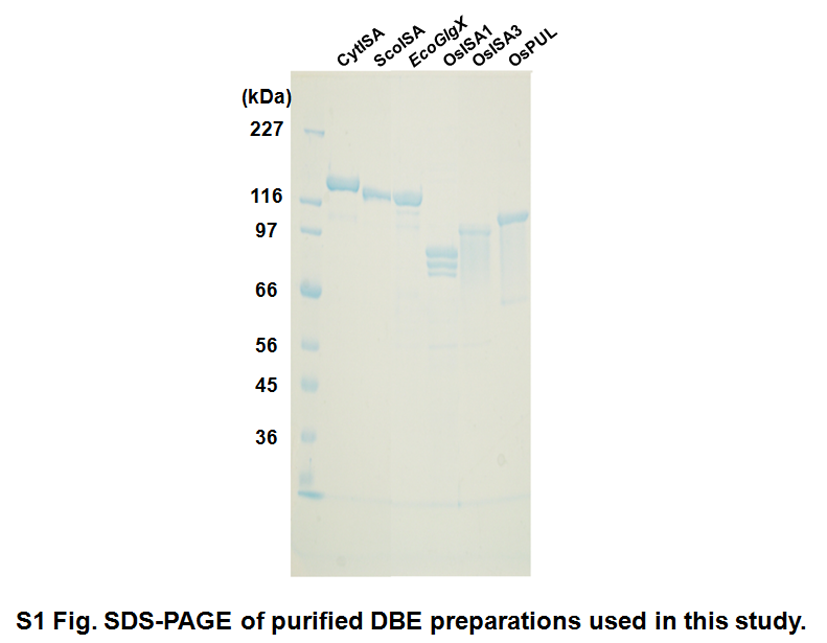

Supplement: S1 Fig — (TIF) [file pone.0157020.s001.tif]

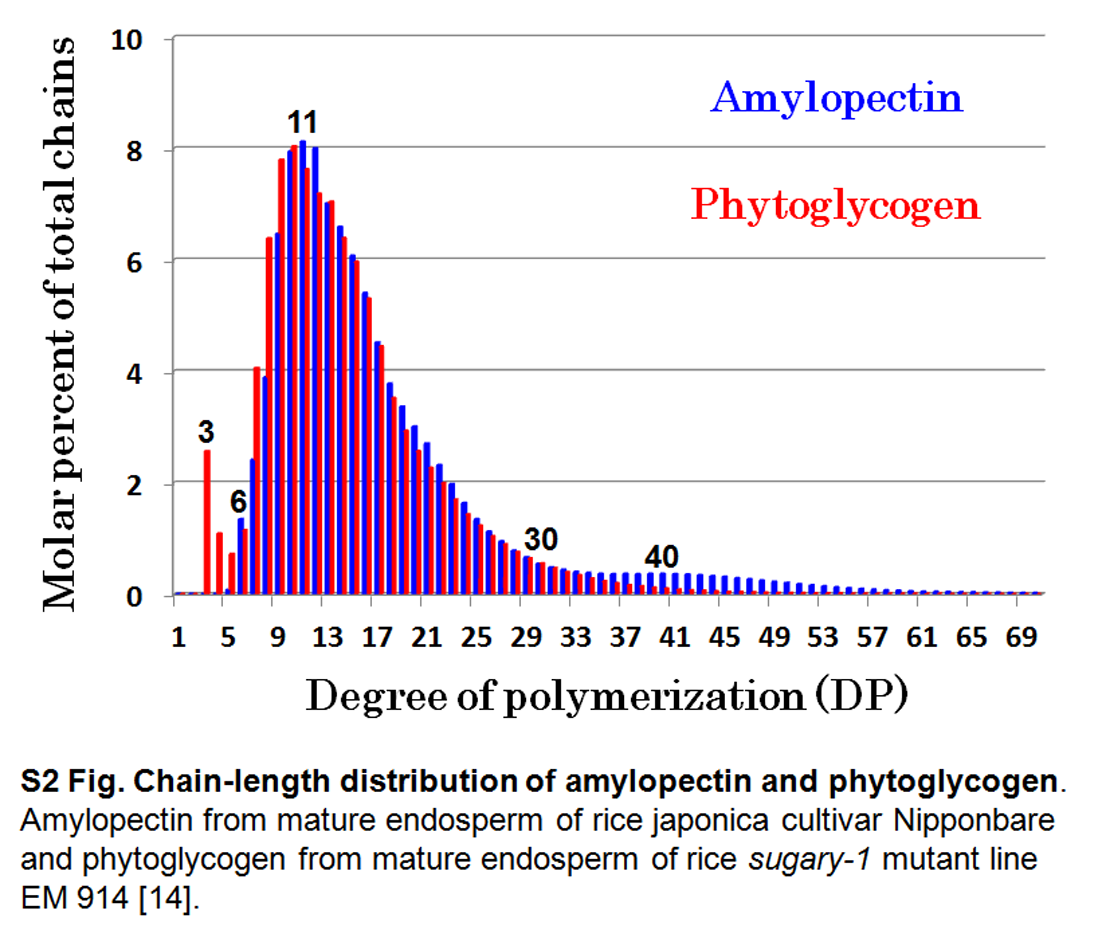

Supplement: S2 Fig — Amylopectin from mature endosperm of rice japonica cultivar Nipponbare and phytoglycogen from mature endosperm of rice sugary-1 mutant line EM 914 [14]. (TIF) [file pone.0157020.s002.tif]

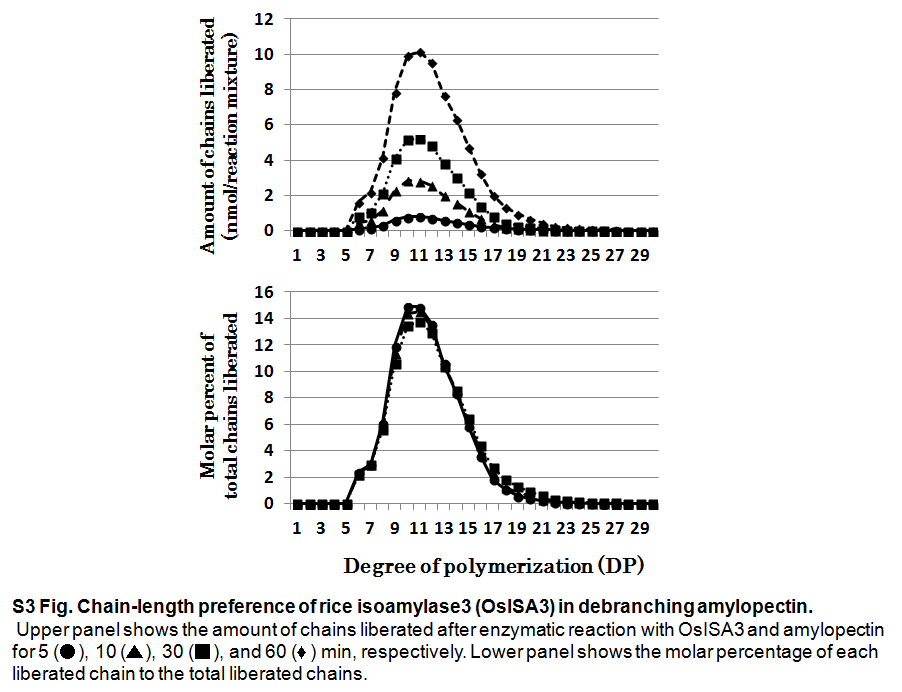

Supplement: S3 Fig — Upper panel shows the amount of chains liberated after enzymatic reaction with OsISA3 and amylopectin for 5 (●), 10 (▲), 30 (■), and 60 (♦) min, respectively. Lower panel shows the molar percentage of each liberated chain to the total liberated chains. (TIF) [file pone.0157020.s003.tif]

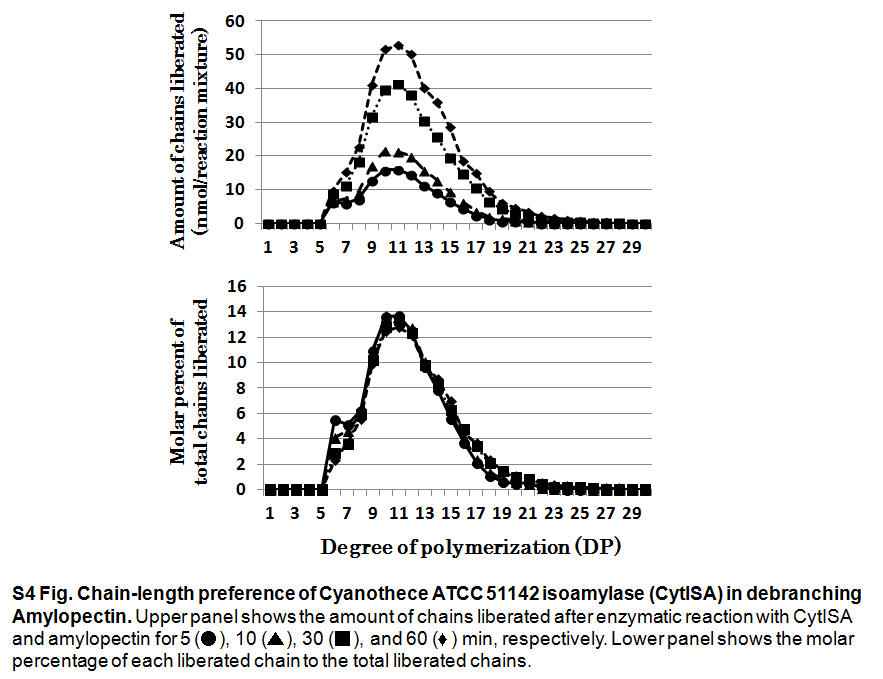

Supplement: S4 Fig — Upper panel shows the amount of chains liberated after enzymatic reaction with CytISA and amylopectin for 5 (●), 10 (▲), 30 (■), and 60 (♦) min, respectively. Lower panel shows the molar percentage of each liberated chain to the total liberated chains. (TIF) [file pone.0157020.s004.tif]

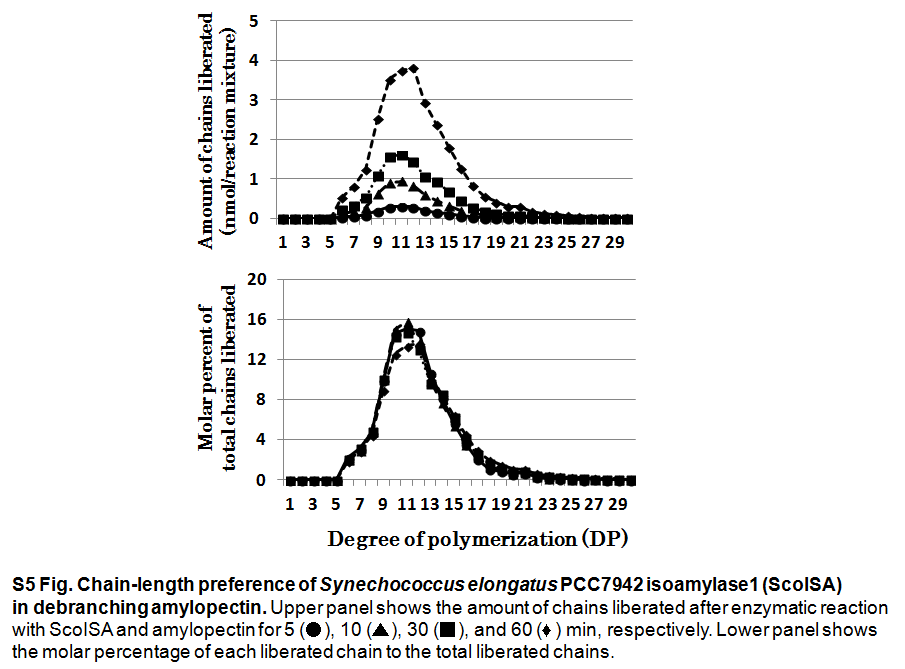

Supplement: S5 Fig — Upper panel shows the amount of chains liberated after enzymatic reaction with ScoISA and amylopectin for 5 (●), 10 (▲), 30 (■), and 60 (♦) min, respectively. Lower panel shows the molar percentage of each liberated chain to the total liberated chains. (TIF) [file pone.0157020.s005.tif]

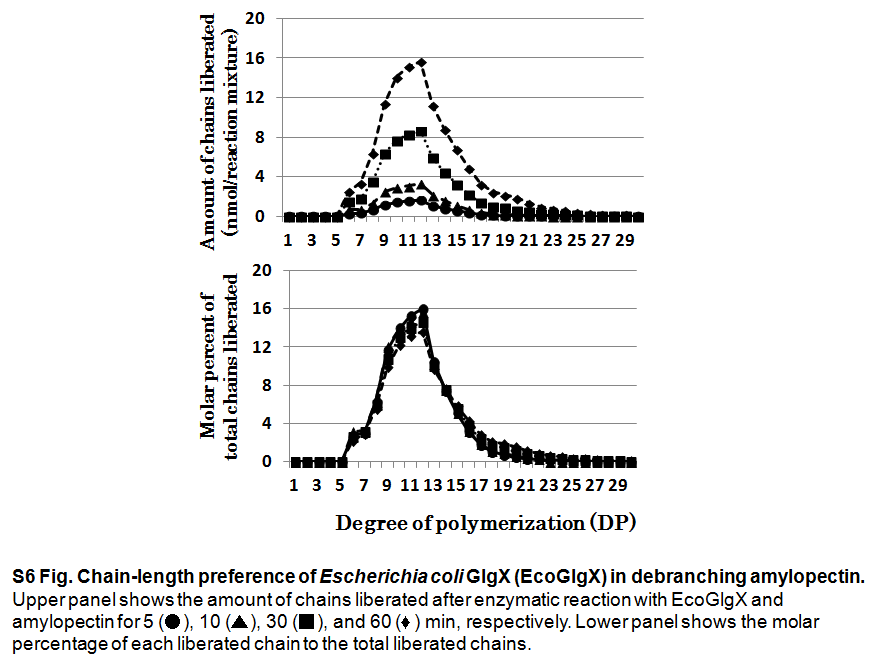

Supplement: S6 Fig — Upper panel shows the amount of chains liberated after enzymatic reaction with EcoGlgX and amylopectin for 5 (●), 10 (▲), 30 (■), and 60 (♦) min, respectively. Lower panel shows the molar percentage of each liberated chain to the total liberated chains. (TIF) [file pone.0157020.s006.tif]

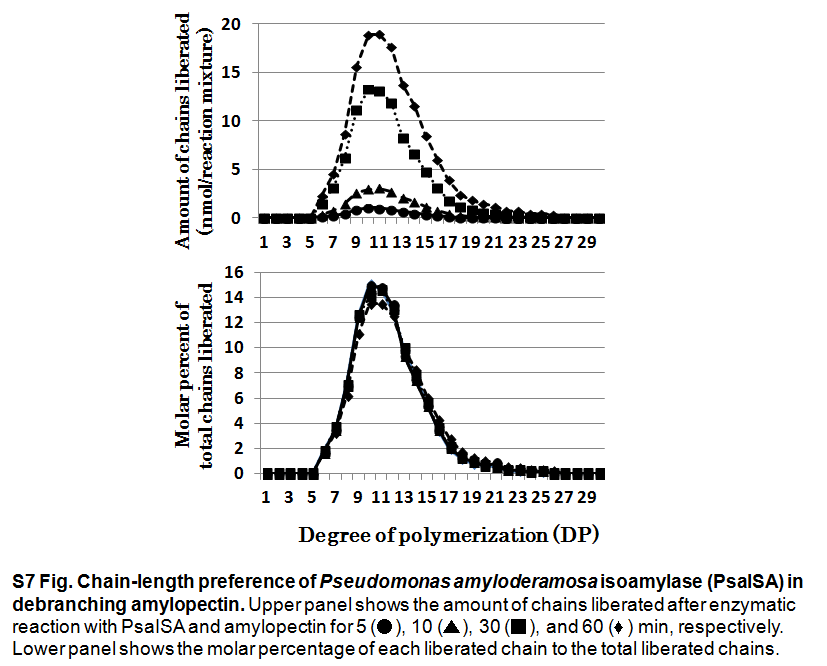

Supplement: S7 Fig — Upper panel shows the amount of chains liberated after enzymatic reaction with PsaISA and amylopectin for 5 (●), 10 (▲), 30 (■), and 60 (♦) min, respectively. Lower panel shows the molar percentage of each liberated chain to the total liberated chains. (TIF) [file pone.0157020.s007.tif]

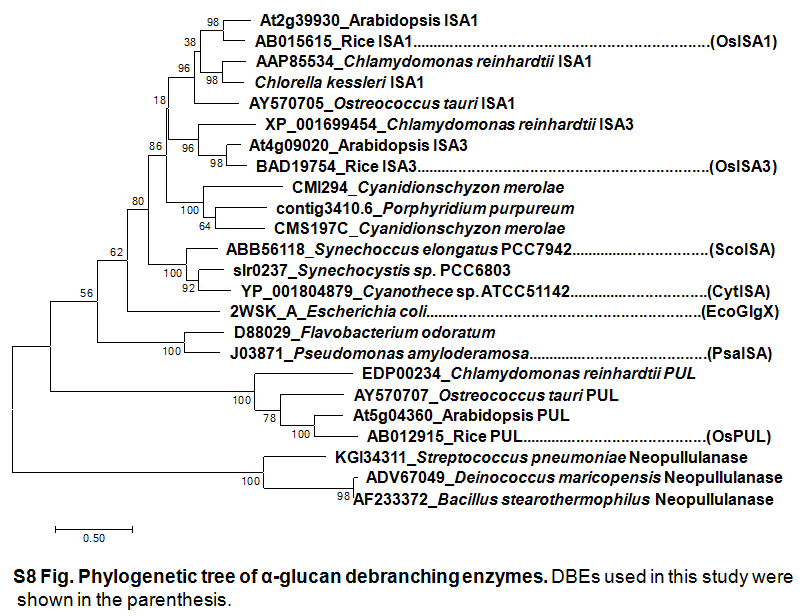

Supplement: S8 Fig — DBEs used in this study were shown in the parenthesis. (TIF) [file pone.0157020.s008.tif]
